# Comparative inhibitory effects of phillyrin and phillygenin on elastase: mechanisms and therapeutic potential

**DOI:** 10.3389/fnut.2026.1723757

**Published:** 2026-02-11

**Authors:** Wenhui Zhang, Jinfeng Fu, Hongliu Yao, Yongxue Li, Jingang Mo, Yi Guan, Yan Wang, Lihao Lin

**Affiliations:** 1 Department of Neurosurgery, The Second Hospital of Tianjin Medical University, Tianjin, China; 2 College of Chemistry, Changchun Normal University, Changchun, China; 3 Department of Neurosurgery, The First Hospital of Jilin University, Changchun, China; 4 School of Life Science, Changchun Normal University, Changchun, China

**Keywords:** elastase, inhibition, lignan compounds, molecular docking, phillygenin, phillyrin

## Abstract

Elastase, a serine protease, has been implicated in chronic obstructive pulmonary disease and systemic inflammatory response syndrome. In this study, we evaluated the effects of phillyrin and phillygenin, 2 major *Forsythia* lignans, on elastase inhibition. Both compounds exhibited competitive inhibition, as confirmed by enzymatic kinetics, spectroscopy, and molecular docking. Phillygenin exhibited stronger activity (IC_50_ 0.5 mmol/L, *K*_i_ 4.0 × 10^−4^ mol/L) than phillyrin (IC_50_ 1.5 mmol/L, *K*_i_ 9.7 × 10^−4^ mol/L), likely due to reduced steric hindrance. Spectroscopic analysis revealed ligand-induced conformational changes in elastase, characterized by increased α-helix and random coil content and decreased β-sheet structures. Docking revealed interactions involving π-cation, π-sigma, hydrogen bonds, hydrophobic forces, electrostatics, and van der Waals effects. These results provide mechanistic insights into the inhibitory effects of phillyrin and phillygenin and highlight their potential as therapeutic agents for elastase-related diseases.

## Graphical Abstract

**Figure:**
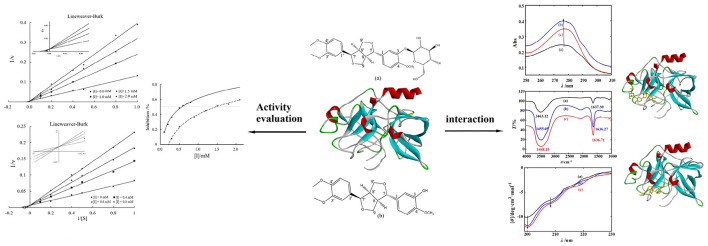

## Highlights

- Phillyrin and phillygenin significantly inhibit elastase through a competitive inhibition mechanism at the active site.
- Phillyrin and phillygenin binding induces conformational changes in elastase.
- Binding of phillyrin and phillygenin to elastase is primarily mediated by hydrogen bonds and various interactions, including π-cation, π-sigma, hydrophobic, electrostatic, and van der Waals forces.
- Phillyrin and phillygenin are promising candidates for therapeutic application in elastase-related inflammatory responses.

## 1 Introduction

Elastase is a destructive serine protease released by polymorphonuclear leukocytes in response to inflammatory stimuli, playing a pivotal role in the initiation and progression of inflammatory disorders (1). The degradation of elastin, a key structural protein that maintains pulmonary and vascular elasticity, causes sustained tissue injury when the physiological balance between elastase and its endogenous inhibitors is disrupted (2). This process is closely implicated in a range of elastase-associated diseases, including chronic obstructive pulmonary disease (COPD), acute respiratory distress syndrome (ARDS), pulmonary fibrosis, and systemic inflammatory response syndrome (3–7).

Although synthetic elastase inhibitors, such as sivelestat, have been developed to treat acute lung injury and ARDS by inhibiting excessive inflammatory responses, their high cost and potential safety concerns limit their widespread clinical application. In addition, conventional anti-inflammatory therapies, including corticosteroids and non-steroidal anti-inflammatory drugs (NSAIDs), are often associated with significant adverse effects during long-term use (8, 9). Consequently, the development of safe, effective, and affordable elastase inhibitors remains an unmet clinical requirement.

Natural products, particularly lignans, have attracted increasing attention as potential elastase inhibitors owing to their low toxicity and favorable bioavailability (10). Several lignans isolated from *Forsythia suspensa*, a traditional medicinal plant widely used for its detoxifying and anti-inflammatory properties, have demonstrated notable elastase inhibition and anti-inflammatory activity (11–14). The plant is officially listed in the Pharmacopeia of the People's Republic of China, where *forsythiaside* A and phillyrin are designated as quality control markers (15).

**Figure 1:**
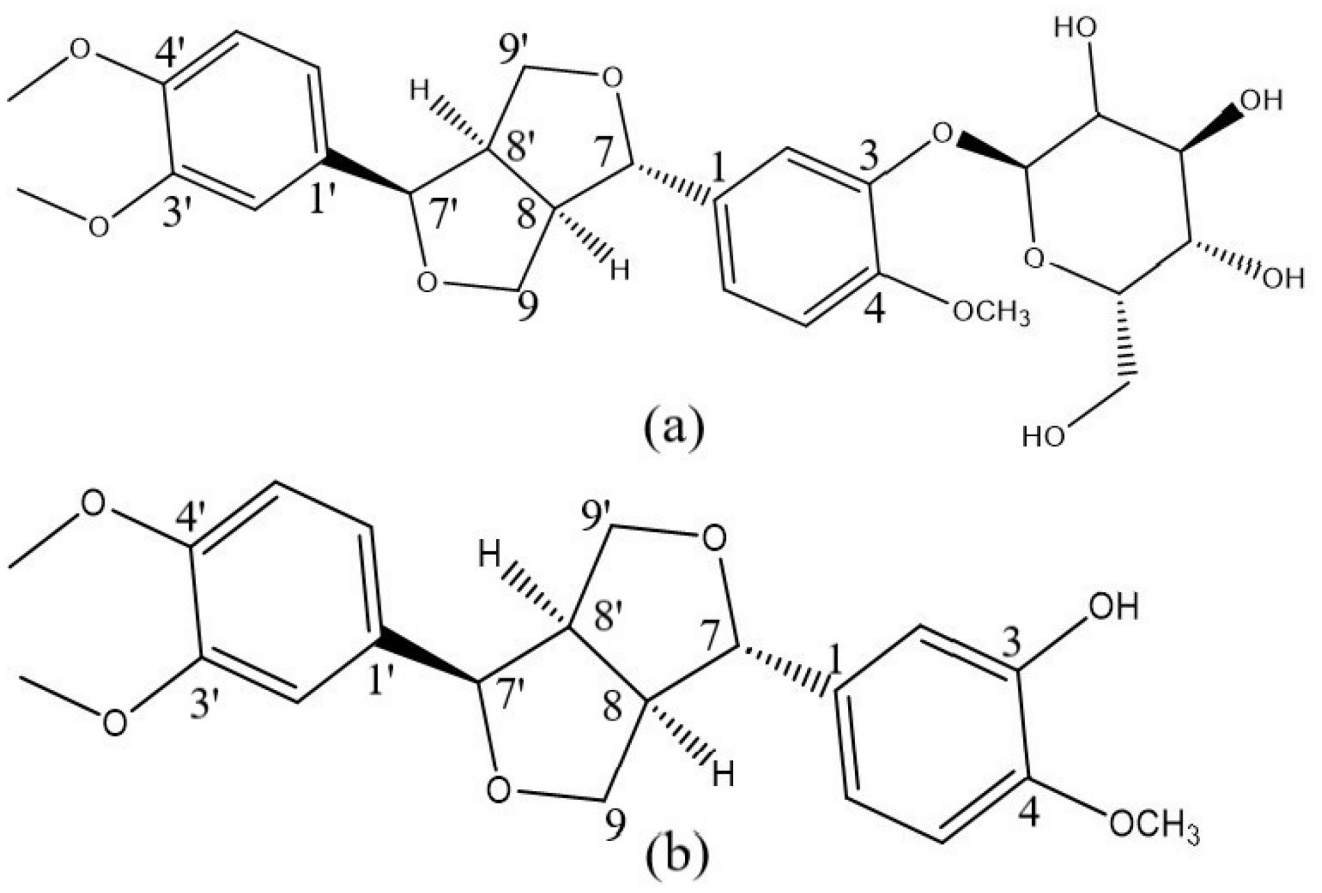
Molecular structures of phillyrin **(a)** and phillygenin **(b)**.

Phillyrin (C_27_H_34_O_11_; Figure 1a), a major lignan constituent of *Forsythia suspensa* (16), and its aglycone phillygenin (C_21_H_24_O_6_; Figure 1b) exhibit a broad spectrum of biological activities (17), including multi-target anti-inflammatory, antiviral, antioxidant, and anticancer effects (18–23). In addition, phillyrin has been reported to exert inhibitory activity against lipases, highlighting its capacity to modulate enzymatic functions beyond inflammatory pathways (24, 25). Structurally, both compounds are classified as dihydrofuran-type lignans containing hydroxyl groups; however, they differ at the third carbon position of the aromatic ring, where phillygenin bears a hydroxyl group, whereas phillyrin is substituted with a glucoside moiety containing 3 hydroxyl groups. These structural features may facilitate their interactions with elastase. Although previous studies have suggested that phillyrin can suppress elastase activity and attenuate *Pseudomonas aeruginosa* infection (26), the precise inhibitory mechanisms of phillyrin and phillygenin against elastase, as well as their structure–activity relationships, remain poorly defined.

In this study, we systematically evaluated the inhibitory effects of phillyrin and phillygenin on elastase activity. Enzyme kinetic analyses were performed to determine IC_50_ values, inhibition types, and *K*_i_ constants. The elastase conformational changes induced by inhibitor binding were investigated using ultraviolet–visible (UV–Vis) spectroscopy, Fourier transform infrared (FT-IR) spectroscopy, and circular dichroism (CD). Furthermore, molecular docking simulations were conducted to elucidate the binding modes and structure–activity relationships. Collectively, these findings provide mechanistic insights into lignan–elastase interactions and support the development of natural elastase inhibitors for the treatment of inflammatory diseases.

## 2 Materials and methods

### 2.1 Materials

Porcine pancreatic elastase (PPE; EC 3.4.21.36) and N-Suc-Ala-Ala-Ala-p-nitroanilide were purchased from Sigma-Aldrich (St. Louis, MO, USA). Phillyrin and phillygenin were purchased from Acmec (Shanghai, China). All the reagents were of analytical grade. Stock solutions were prepared in phosphate-buffered saline (PBS; 0.01 M, pH 6.86) and diluted to the required concentrations prior to use.

### 2.2 Apparatus

The experiments were performed using a FlexStation 3 microplate reader (Molecular Devices, USA), a Cary 300 UV–Vis spectrophotometer (Agilent Technologies, USA), a Nicolet iS50 FT-IR spectrometer (Thermo Fisher Scientific, USA), and a MOS-500 CD spectrometer (Bio-Logic, France).

### 2.3 Methods

#### 2.3.1 PPE inhibition activity assay

PPE inhibition activity was determined using N-Suc-Ala-Ala-Ala-p-nitroanilide as the substrate. Phillyrin and phillygenin served as the positive controls. PPE, substrate, and inhibitors were prepared in PBS (pH 6.86). PPE (1 μM, 25 μL) was incubated with phillyrin or phillygenin at various concentrations in a 96-well plate for 2 min at room temperature, followed by the addition of substrate (1 mM, 25 μL). The final reaction volume was 250 μL. Enzymatic activity was monitored at 405 nm using a microplate reader. PBS was used as a negative control.

The inhibition rate was calculated according to the following equation (23):

$$\begin{array}{l} \text{Inhibition rate }\left(\%\right)\;=\left[\left(v_{A}-v_{B}\right)/{\text{v}}_{\text{A}}\right]\;\times \;100 \end{array}$$

where v_A_ and v_B_ are the reaction rates of the control and inhibitor-treated samples, respectively.

#### 2.3.2 Kinetic analysis for competitive-type inhibition

Kinetic inhibition assays were conducted by incubating PPE (1 μM, 25 μL) with increasing concentrations of phillyrin (0, 1, 1.5, and 2 mM) or phillygenin (0, 0.4, 0.6, and 0.8 mM). Substrate concentrations ranged from 10 to 100 mM, with a fixed final reaction volume of 250 μL. The reaction rates were recorded at 405 nm. The enzymatic reaction rate was monitored at 405 nm using a microplate reader, with PBS serving as a negative control. The inhibition constant (*K*_i_) and mechanism were evaluated using Lineweaver–Burk plots, where *v* represents the reaction rate, *K*_m_ is the Michaelis–Menten constant, [*I*] is the inhibitor concentration, [*S*] is the substrate concentration, and *V*_m_ is the maximum reaction rate obtained by varying the substrate concentration. The apparent Michaelis–Menten constant ($K_{\text{m}}^{\prime }$) was calculated, and a linear fit of the secondary plots of $K_{\text{m}}^{\prime }$ vs [I] was performed, assuming a single inhibition site (27–29).

$$\begin{array}{l} \frac{1}{\text{v}}=\frac{K_{\text{m}}}{{\text{v}}_{\text{m}}\left[\text{S}\right]}+\frac{1}{{\text{v}}_{m}} \end{array}$$

The Lineweaver-Burk double reciprocal method involves plotting the inverse of the substrate concentration on the *x*-axis and the inverse of the enzyme reaction rate on the *y*-axis, with different inhibitor concentrations as parameters. The *K*_i_ was calculated using the following equation:

$$\begin{array}{l} {K^{\prime }}_{m}=K_{m}\left(1+\frac{\left[I\right]}{K_{i}}\right) \end{array}$$

#### 2.3.3 UV-Vis spectra measurements

The UV–Vis absorption spectra of PPE and PPE–ligand complexes were recorded at room temperature using an Ultrospec 4000 UV–Vis Pharmacia spectrophotometer. PPE (10 μM) was incubated with phillyrin or phillygenin (50 μM) for 5 min prior to scanning. Spectra were obtained over a wavelength range of 190–800 nm, using PBS as a blank.

#### 2.3.4 FT-IR spectra measurements

FT-IR spectra of PPE in the absence and presence of phillyrin or phillygenin were recorded at room temperature over the range of 4,000–1,000 cm^−1^. PPE concentration was fixed at 5.0 μM, with an inhibitor-to-enzyme molar ratio of 2:1. The buffer and free ligand spectra were recorded separately and digitally subtracted from each other.

#### 2.3.5 CD spectra measurements

CD spectra were recorded between 195 and 280 nm under a nitrogen atmosphere at room temperature using a 1 mm quartz cuvette. PPE concentration was 5.0 μM, and the inhibitor-to-enzyme molar ratio was set to 2:1. All the spectra were corrected for buffer signals. Secondary structure content (α-helix, β-sheet, random coil) was analyzed from the CD data using the SELCON3 program.

#### 2.3.6 Molecular docking studies

Molecular docking simulations were performed using AutoDock software to investigate the binding interactions between porcine pancreatic elastase (PPE), phillyrin, and phillygenin. Flexible ligand docking was performed by treating the protein structure as rigid. The PPE crystal structure (PDB ID: 9EST; resolution 1.9 Å) was prepared using the Protein Preparation Wizard in Schrödinger, including protonation and correction of incomplete amino acid residues. The three-dimensional structures of PPE, phillyrin, and phillygenin were energy-minimized using ChemBio 3D Ultra 14.0 prior to docking. Docking calculations were conducted using AutoGrid software to generate energy-scoring grid maps with a grid spacing of 4.0 Å. Fifty independent docking runs were performed for each ligand, and the binding conformation with the lowest predicted binding energy was selected as the most favorable. All the other AutoDock parameters were set to their default values (30).

### 2.4 Statistical analyses

All experiments were performed in triplicate, and data are presented as mean ± standard deviation (SD). Statistical analyses were conducted using SPSS 27.0 and Origin 2024. Differences among groups were evaluated by one-way ANOVA, with *P* < 0.05 considered statistically significant.

## 3 Results

### 3.1 Inhibition of PPE by phillyrin and phillygenin

PPE and human leukocyte elastase (HLE) exhibit consistent activities and high structural similarities in crystallographic studies. PPE is a crucial enzyme in the serine protease family and is composed of 240 amino acids with a molecular weight of 26 kDa (31). Its active site contains 214 serine, 71 histidine, and 119 aspartic acid residues (32). PPE exhibits broad hydrolytic activity and is capable of breaking down not only insoluble elastin, but also proteins such as casein, hemoglobin, and albumin, making it a versatile endopeptidase (33, 34). Because PPE is more readily available, it serves as an excellent model for HLE.

**Figure 2:**
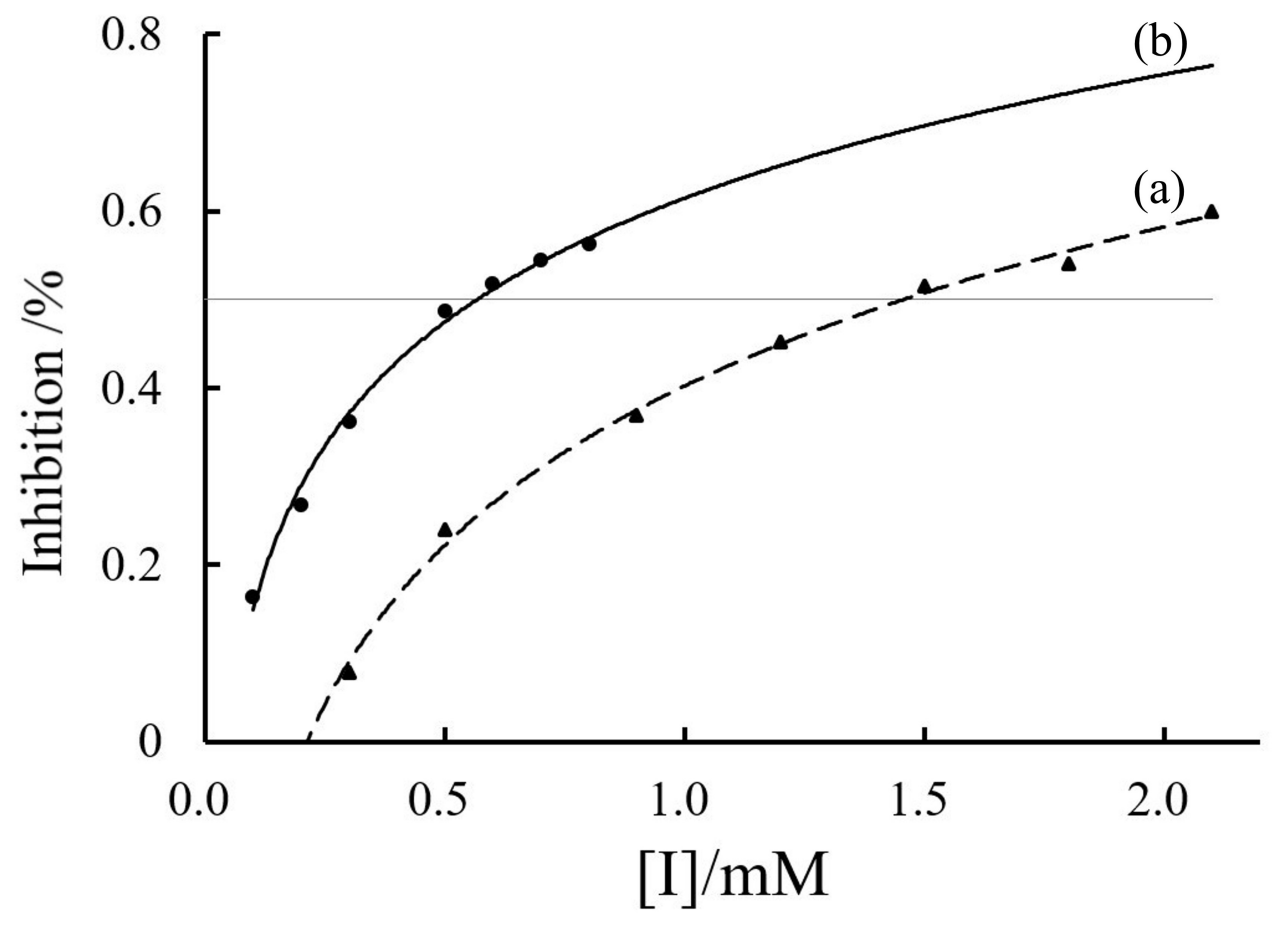
PPE inhibitory activities of phillyrin (a) and phillygenin (b).

As illustrated in Figure 2, both phillyrin and phillygenin inhibited elastase activity in a dose-dependent manner. Phillyrin exhibited an IC_50_ of 1.5 mmol/L, indicating that this concentration reduced elastase activity by 50%. In contrast, phillygenin demonstrated a stronger inhibitory effect, with an IC_50_ of 0.5 mmol/L, signifying that this concentration also results in 50% inhibition of elastase activity. These IC_50_ values reflect the effectiveness of each compound as an elastase inhibitor (35, 36), which is consistent with previous findings. For instance, Ping-Chung Kuo et al. (12) reported a significant reduction in elastase levels when forsythoside was administered at concentrations between 0.0625 and 0.25 mg/ml. Although both compounds effectively inhibited elastase activity, phillygenin consistently outperformed phillyrin at all the concentrations tested. This variation in potency may stem from structural differences between the 2 molecules; specifically, the presence of a glycoside group in phillyrin may affect its binding interactions with PPE. Conversely, phillygenin, which lacks this glycoside moiety, likely interacts more favorably with the enzyme, leading to enhanced inhibitory effects. These findings suggest that both phillyrin and phillygenin are promising candidates for further investigation as elastase inhibitors, with phillygenin showing particular promise due to its high potency. The structural differences between these compounds and their implications for elastase inhibition warrant further exploration to better understand their mechanisms of action.

### 3.2 Types of phillyrin and phillygenin inhibition on PPE

**Figure 3:**
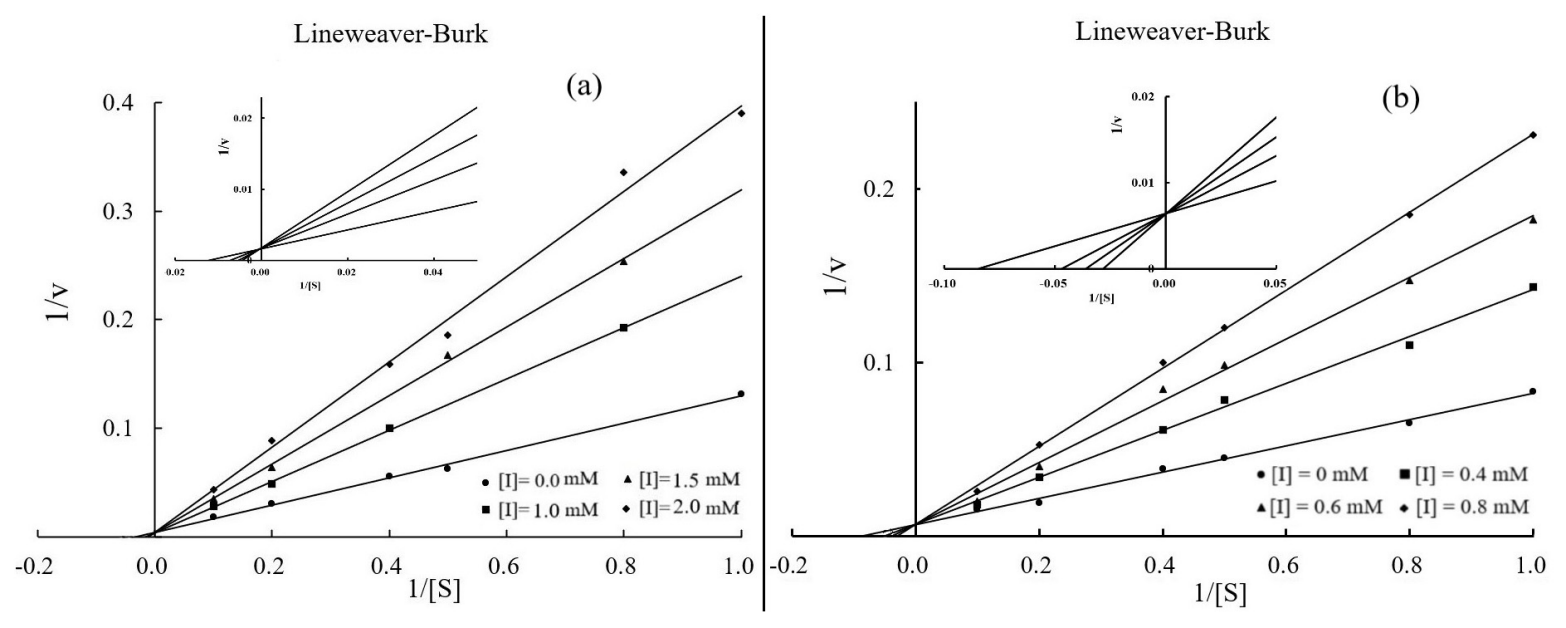
Lineweaver-Burk plots of phillyrin **(a)** and phillygenin **(b)**.

Figure 3 illustrates the inhibition kinetics of phillyrin and phillygenin against elastase activity as analyzed using Lineweaver–Burk plots (29). K_i_ indicates the strength of the effect of an inhibitor on the target enzyme, with lower values indicating stronger inhibition. The data demonstrated that as the concentrations of phillyrin and phillygenin increased, the slope of the Lineweaver–Burk plot also increased, while the *y*-intercept remained constant. This pattern suggested an increase in the K_m_, whereas the V_m_ remained unchanged, confirming that both phillyrin (Figure 3a) and phillygenin (Figure 3b) exhibited competitive elastase inhibition. During competitive inhibition, the inhibitor competes with the substrate for the active sites of the enzyme. The K_m_ and K_i_ values of both inhibitors were derived from double-reciprocal plots. Specifically, phillyrin had a K_m_ of 7.14 mmol/L, and a K_i_ of 9.7 × 10^−4^ mol/L, while phillygenin exhibited a K_m_ of 11.8 mmol/L and a K_i_ of 4.0 × 10^−4^ mol/L. The lower K_i_ value for phillygenin indicated stronger competition for the active site of elastase and greater inhibitory potency than phillyrin. The enhanced inhibitory strength of phillygenin may be attributed to its molecular structure, which undergoes significant conformational changes upon binding to the enzyme.

### 3.3 UV–Vis absorption spectra of the effects of phillyrin and phillygenin on PPE

**Figure 4:**
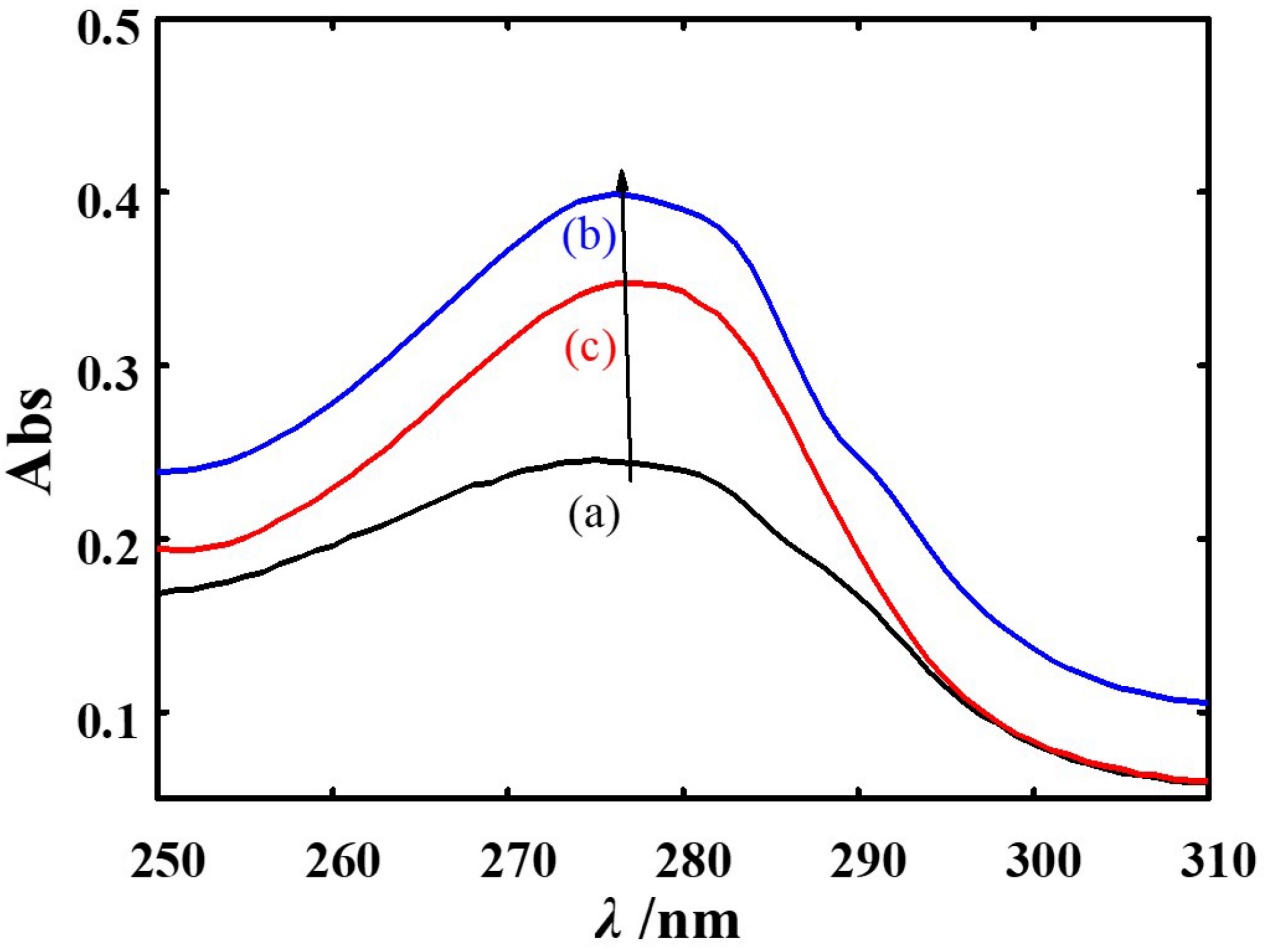
UV-vis spectra of PPE (a), phillyrin-PPE (b), and phillygenin-PPE (c).

Figure 4 shows that the UV–Vis absorption spectra of PPE changed upon the addition of phillyrin and phillygenin, primarily because of the conjugated double bonds of tyrosine (Tyr) residues and the indole rings of tryptophan (Trp) residues (see curve a). However, the UV absorption of Trp was stronger than that of Tyr, resulting in a peak at 280 nm, which was predominantly attributable to Trp (37–39). Notably, the absorption values of the mixtures (A_PPE_ + A_phillyrin_ and A_PPE_ + A_phillygenin_) differed from the sum of the individual absorption spectra of PPE and each ligand (A_phillyrin − PPE_ and A_phillygenin − PPE_). This discrepancy indicates a significant interaction between the ligands and PPE. In addition, the absorption peak at 280 nm exhibited a blue shift following the addition of phillyrin and phillygenin, suggesting conformational changes in PPE. This blue shift indicates that phillyrin and phillygenin interact directly with the Trp residues of PPE, which are endogenous chromophores (40). This interaction likely alters the microenvironment of these residues, resulting in increased overall polarity of the protein. Consequently, the hydrophobicity of the protein decreases, implying a conformational change in PPE upon binding to phillyrin and phillygenin.

### 3.4 FT-IR spectra of the effects of phillyrin and phillygenin on PPE

**Figure 5:**
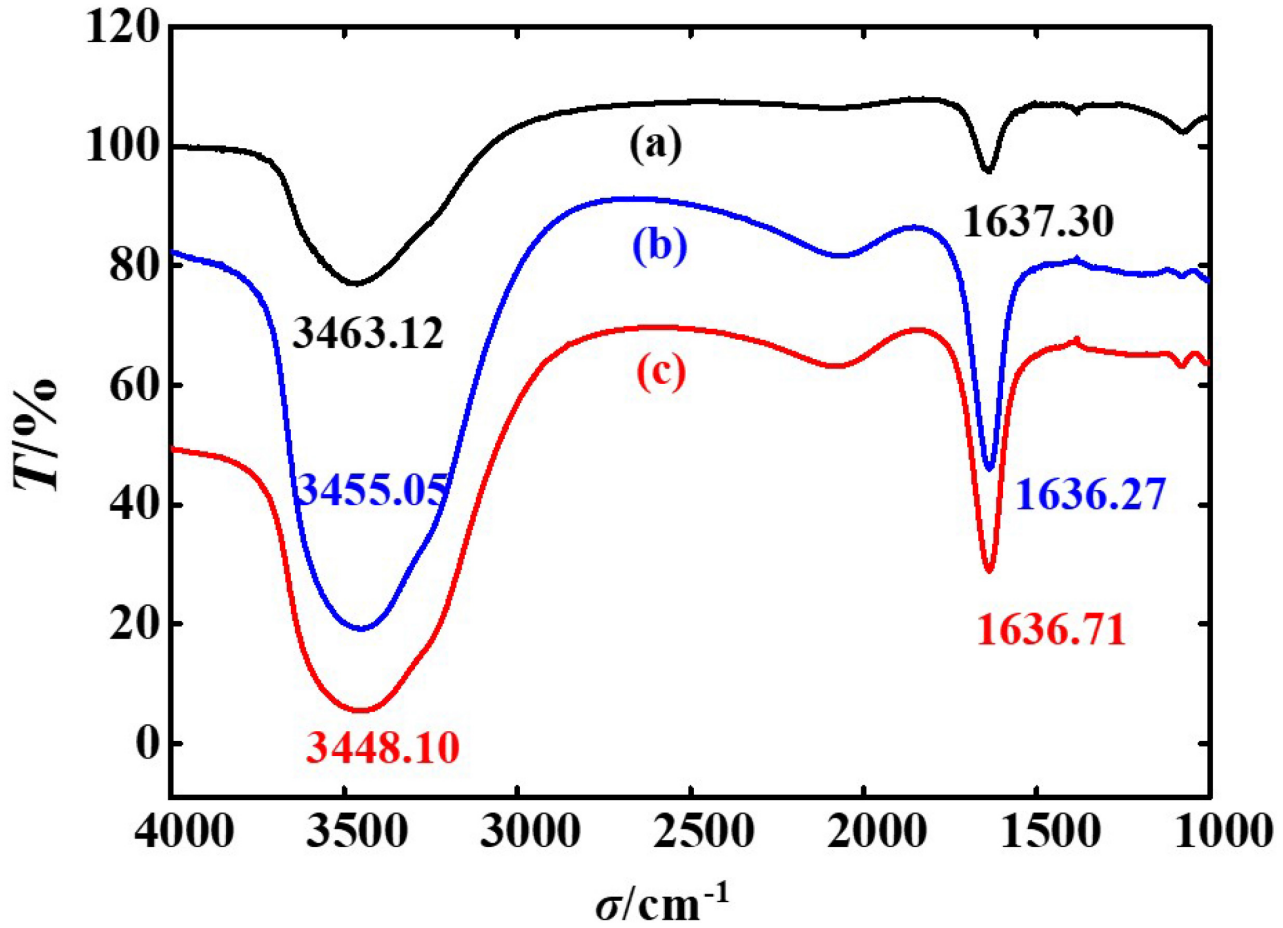
FT-IR spectra of PPE (a), phillyrin-PPE (b), and phillygenin-PPE (c).

The amide I band in the FT-IR spectrum serves as a valuable indicator for analyzing protein secondary structures, because distinct secondary structures exhibit specific vibrational frequencies. Figure 5 shows FT-IR spectra demonstrating the interactions between phillyrin, phillygenin, and PPE. Within the spectral range of 1,600–1,700 cm^−1^, a significant stretching vibration of the carbonyl (C=O) group can be detected (41). When phillyrin and phillygenin were introduced, this band shifted to lower wavenumbers, which was likely attributable to the coupling effect between the amide hydrogen bonds and the instantaneous dipole moments created by the ligands (42). These interactions could lead to splitting of the amide I band, resulting in changes in both the distance and angle of the dipole moments linked to the amide bonds.

Additionally, the characteristic absorption peak of the N–H bond on the indole ring of Trp, typically found in the 3,400–3,500 cm^−1^ region (43), also shifts to lower wavenumbers after the addition of phillyrin and phillygenin. This change suggests that the ligands weaken the N–H bond strength of Trp, resulting in a decrease in the stretching vibration frequency, and consequently, a shift to lower wavenumbers. These observations provide insights into the conformational changes induced in PPE upon interaction with phillyrin and phillygenin. Shifts in the amide I band and N–H bond vibrations indicated that the binding of these ligands may alter the hydrogen-bonding environment, affecting the secondary structure of elastase. These structural modifications may influence the enzymatic activity of PPE and potentially contribute to the inhibitory effects of phillyrin and phillygenin.

### 3.5 CD spectra of the effects of phillyrin and phillygenin on PPE

**Figure 6:**
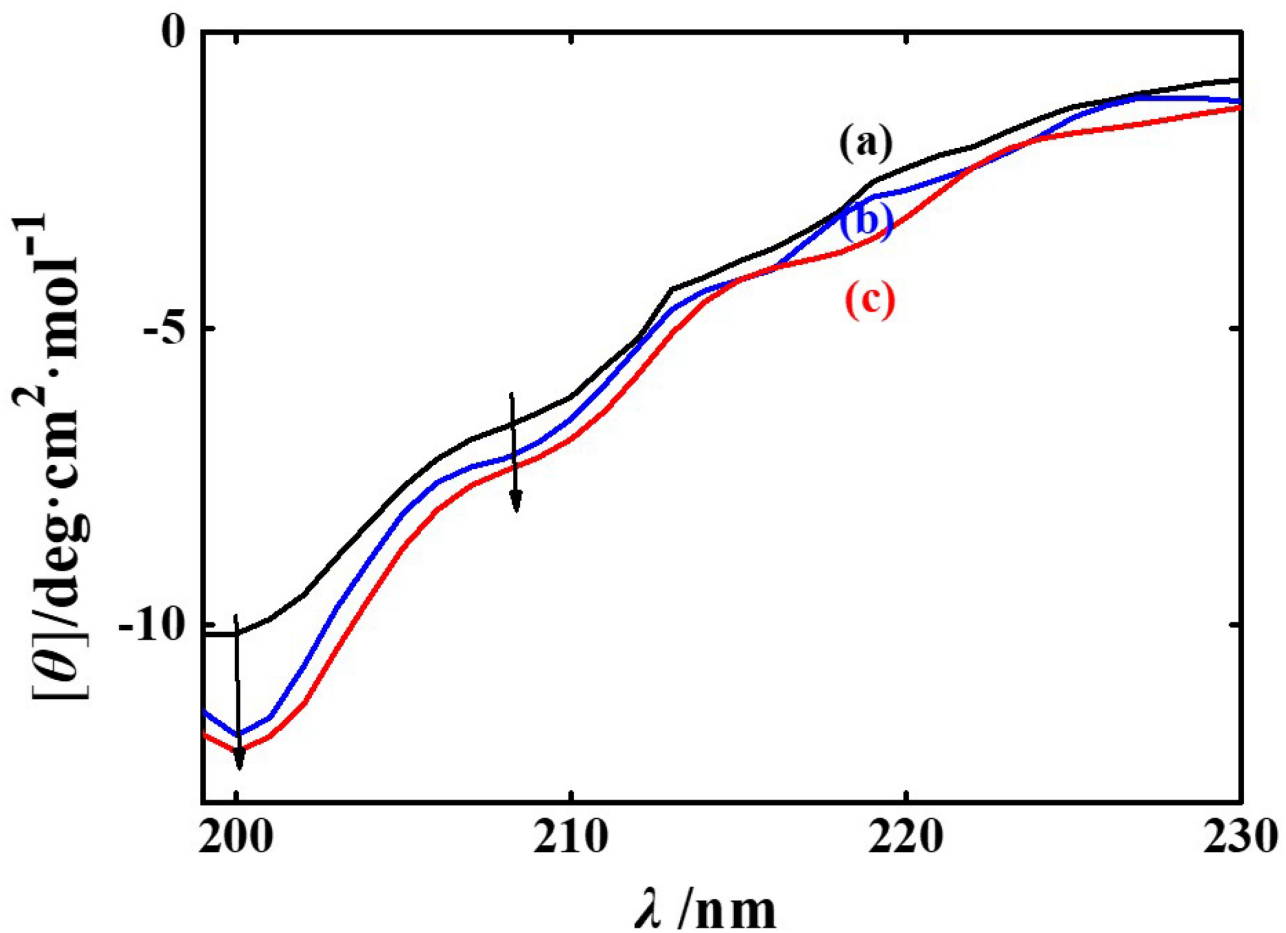
The CD spectra of PPE (a), phillyrin-PPE (b), and phillygenin-PPE (c).

Figure 6 shows a comprehensive analysis of the conformational changes in PPE, as assessed by CD spectral measurements, which are particularly sensitive to modifications in the secondary structure of the protein. The characteristic peaks of the CD spectra in the far-ultraviolet region (185–245 nm) reveal alterations in the main-chain conformation of protein polypeptides and the surrounding chromophore microenvironment (44, 45). The CD spectrum of PPE in isolation displays two negative peaks at 208 and 200 nm, which are indicative of the presence of α-helix and random coil structures, respectively (46, 47).

Upon addition of phillyrin and phillygenin to PPE, enhancement of the CD signal was observed. Analysis of the CD data revealed that the proportion of α-helix increased from 5.20% to 5.29% and 5.43%, respectively, while the proportion of β-sheet structures decreased from 52.80% to 50.54% and 49.28% (Table 1). These findings suggested that the interactions between phillyrin and phillygenin and the polypeptide chains of PPE led to significant modifications in the hydrogen-bond network, altering the secondary structure of the protein. In addition, phillygenin exerted a greater effect on the secondary structure of PPE than phillyrin did.

**Table 1:** The contents of secondary structures of free PPE, phillyrin-PPE, and phillygenin-PPE systems.

**System**	**α-helix/%**	**β-fold/%**	**random coil/%**
PPE	5.20	52.80	42.00
Phillyrin-PPE	5.29	50.54	44.17
Phillygenin-PPE	5.43	49.28	45.29

The increase in α-helix content implies that the interaction with these ligands may result in a more compact protein structure. This conformational shift hinders substrate access to the PPE active site, ultimately reducing the enzymatic activity. Concurrently, the decrease in β-sheet structures indicates that the active site of PPE becomes less readily formed, further diminishing substrate binding likelihood. This structural change correlated with the observed decrease in catalytic efficiency, reinforcing the conclusion that phillyrin and phillygenin modulate PPE conformation and activity.

### 3.6 Molecular docking study

**Figure 7:**
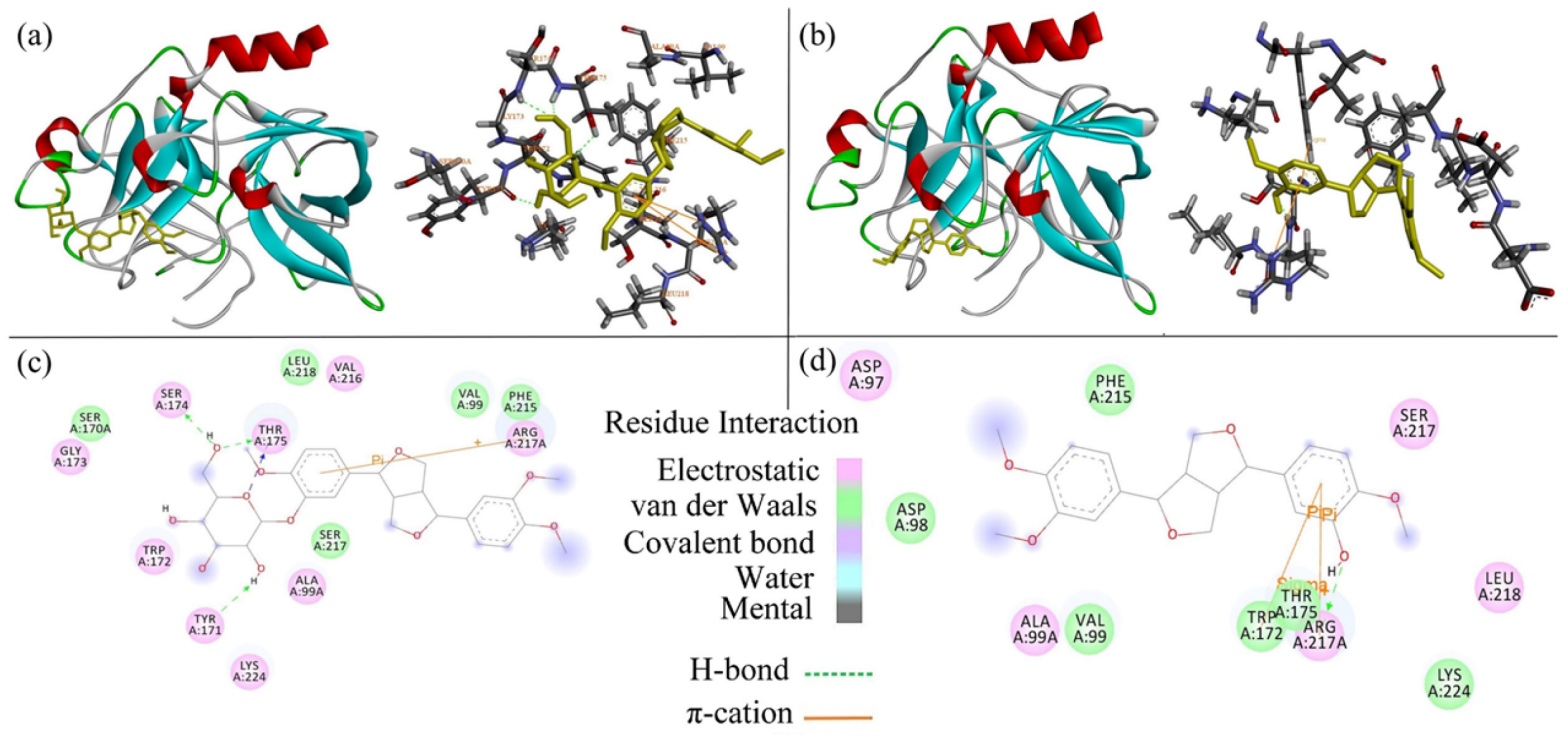
Mechanism of interaction between phillyrin, phillygenin (yellow) and PPE protein (red oxygen, blue nitrogen, and white hydrogen). **(a)** Docking results of 3D map of the best binding site of phillyrin/PPE. **(b)** 3D map of the best binding site of phillygenin/PPE. **(c)** Two-dimensional interaction map of the phillyrin/PPE within the range of ligand 4Å. **(d)** Two-dimensional interaction map of the phillygenin/PPE within the range of ligand 4Å. The dashed lines (green) represent hydrogen-bonding interactions. The solid lines (orange) represent π-cation interactions.

Molecular docking analysis was performed to investigate the binding modes of phillyrin and phillygenin to PPE. As shown in Figure 7a, the optimal docking conformation of phillyrin with PPE exhibited a predicted binding energy of−7.4 kcal/mol. Similarly, the best-ranked docking pose of phillygenin (Figure 7b) showed a binding energy of −7.1 kcal/mol, indicating favorable ligand–enzyme interactions under computational conditions.

To ensure reproducibility, docking simulations were conducted using AutoDock with explicitly defined grid-box parameters. For phillyrin, the grid box was centered at (center_*x* = −5.934, center_*y* = 27.480, and center_*z* = 43.081) with dimensions of 48 × 40 × 40 Å3, using 20 output poses, an energy range of 5 kcal/mol, and an exhaustiveness value of 100. For phillygenin, the grid box center was set at (center_*x* = −6.806, center_*y* = 26.944, and center_*z* = 43.444) with dimensions of 46 × 40 × 44 Å3, and 200 docking poses were generated using the same exhaustiveness and energy range settings. These parameters ensured adequate sampling of the catalytic pocket region surrounding active site residues.

LigPlot analysis (Figures 7c, d) revealed that the 7-benzyl benzene rings of both phillyrin and phillygenin formed π-cation interactions with Arg217A. In addition, phillyrin formed hydrogen bonds with Ser174, Thr175 (2 bonds), and Tyr171, whereas phillygenin formed hydrogen bonds with Arg217A. Notably, phillygenin also exhibited a π-σ interaction with Trp172 (Table 2), suggesting a distinct interaction pattern compared with phillyrin.

**Table 2:** Interaction types of phillyrin or phillygenin on PPE.

**Interaction types**	**Phillyrin**	**Phillygenin**
π-cation	ARG217	ARG217
π-Sigma		TRP172
Hydrogen bonding	SER174, THR175 (2), TYR171	ARG217
Hydrophobic interaction	THR175, VAL99, ARG217, PHE215, TRP172	THR175, VAL99, ASP97, ARG217, TRP172,
Electrostatic interaction forces	GLY173, SER174, ARG217, TRP172, ALA99A, TYR171, LYS224 THR175, VAL216	ASP97, ALA99A, ARG217, SER217, LEU218
Van der waals force	LEU218, SER170A, VAL99, PHE215, SER217	ASP98, PHE215, VAL99, TRP172, THR175, LYS224

## 4 Discussion

Based on the above findings, both phillyrin and phillygenin markedly inhibited PPE activity in a dose-dependent manner, with phillygenin showing greater potency. Combined kinetic, spectroscopic, and molecular docking analyses were performed to elucidate underlying mechanisms. These results consistently demonstrated that both lignans act as competitive inhibitors that interact directly with the catalytic pocket of PPE and induce conformational alterations, ultimately reducing enzyme activity.

### 4.1 Inhibitory potency and kinetic characteristics

Kinetic analysis revealed a typical competitive inhibition pattern for both compounds, as indicated by the Lineweaver– Burk plots, where the slopes increased with inhibitor concentration while the intercepts remained constant. This competitive inhibition pattern is consistent with previous reports describing elastase inhibition by plant-derived polyphenols and lignans, which frequently interact with the catalytic pocket rather than the allosteric sites (48). Similar competitive behaviors have been reported for lignans isolated from *Forsythia suspensa* and other medicinal plants, supporting the notion that phenolic compounds preferentially target the active sites of serine proteases (49).

Phillygenin exhibited a lower K_i_ (4.0 × 10^−4^ mol/L) and IC_50_ value (0.5 mmol/L) than phillyrin (K_i_ = 9.7 × 10^−4^ mol/L; IC_50_ = 1.5 mmol/L), indicating stronger binding affinity and inhibitory potency. Interestingly, although previous studies have suggested that glycosylation may enhance the aqueous solubility and bioavailability of lignans (50), our results indicate that the presence of a bulky glycoside moiety in phillyrin reduces its PPE inhibitory efficiency. This discrepancy may arise from differences in the assay systems, target enzymes, or experimental conditions, highlighting the importance of steric accessibility to the elastase active site in determining inhibitory potency. Structurally, phillyrin and phillygenin differ primarily in the C-3 position of the aromatic ring, where the glucoside moiety in phillyrin is replaced by a single hydroxyl group. This simplification reduces the steric hindrance and may facilitate more efficient accommodation within the narrow catalytic pocket of PPE. However, the bulky glycoside in phillyrin may limit access to the hydrophobic regions of the active site. In contrast, the bulkier glycosidic moiety of phillyrin may restrict access to the hydrophobic region of the active site.

### 4.2 Spectroscopic evidence of conformational alterations

Spectroscopic analyses further supported ligand-induced conformational rearrangements in PPE. UV–Vis spectra revealed a blue shift near 280 nm, attributable to Trp residues, suggesting changes in their microenvironment. Comparable spectral shifts have been reported for elastases interacting with small-molecule inhibitors and phenolic compounds, where ligand binding alters the polarity of the surrounding aromatic residues and perturbs local folding near the active site (51). Therefore, our findings are consistent with previously described elastase–inhibitor systems and support a conserved mechanism of ligand-induced micro environmental remodeling.

FT-IR and CD analyses further indicated alterations in secondary structure, including a modest increase in α-helix content and a reduction in β-sheet structures. Such structural rearrangements have been proposed in earlier studies to restrict substrate diffusion into the catalytic cleft, thereby complementing competitive inhibition (52). The more pronounced spectroscopic changes observed with phillygenin are consistent with its stronger inhibitory potency and suggest a greater capacity to modulate the conformational dynamics of elastase.

It should be emphasized that spectroscopic techniques provide indirect evidence of ligand–enzyme interactions and conformational changes rather than quantitative binding parameters. To further substantiate these findings, surface plasmon resonance (SPR) and related kinetic analyses (e.g., determination of K_D, k_on, and k_off values) will be pursued in future studies to directly quantify the binding affinity and interaction dynamics. Such approaches will critically validate the mechanistic hypotheses proposed here and strengthen the translational relevance of spectroscopic observations.

### 4.3 Molecular docking insights

Molecular docking was employed to elucidate the binding modes of phillyrin and phillygenin within the catalytic pocket of PPE and to provide structural support for their experimentally observed inhibitory effects (53, 54). Similar to recent induced-fit docking studies that emphasized residue-specific interactions underlying enzyme inhibition, our analysis focused on identifying key non-covalent interactions that contribute to ligand stabilization and functional interference.

The best-ranked docking poses showed that both phillyrin and phillygenin were well accommodated within the substrate-binding pocket of PPE, partially overlapping with the catalytic region, consistent with their competitive inhibition profiles. The predicted binding energies indicated favorable ligand–enzyme interactions under computational conditions, although these values primarily reflected relative binding stability rather than physiological potency.

Detailed interaction analysis revealed that both ligands form π-cation interactions between their benzyl aromatic rings and Arg217A, a residue previously implicated in substrate recognition and inhibitor binding in elastase (55, 56). Such aromatic–cation interactions have been frequently reported for competitive elastase inhibitors and are considered important contributors to binding stabilization within the active site (57, 58). In addition, the proximity of aromatic Trp residues (notably Trp172) to the docked ligands provided a structural basis for the experimentally observed UV-Vis spectral changes, suggesting ligand-induced perturbations in the local aromatic microenvironment.

Phillyrin forms multiple hydrogen bonds with Ser174, Thr175, and Tyr171, indicating strong polar contact with the surrounding active-site residues. However, despite forming fewer hydrogen bonds, phillygenin exhibited an additional π-σ interaction with Trp172, which may enhance hydrophobic packing and promote a more compact ligand–enzyme complex. Similar observations have been reported in structure-based inhibitor studies, where hydrophobic and aromatic interactions, rather than the hydrogen bond number alone, have been shown to critically influence inhibitory potency (57, 59, 60).

The apparent discrepancy between the hydrogen bond quantity and experimental inhibition strength underscores that effective elastase inhibition depends on a combination of steric compatibility, ligand flexibility, and hydrophobic interactions (60, 61). The absence of a glycosyl moiety in phillygenin reduces steric hindrance and likely allows more efficient accommodation within the narrow catalytic pocket of PPE, thereby facilitating optimal positioning relative to the key functional residues and more effective competition with the substrate.

Taken together, these docking results provide a mechanistically coherent structural explanation for the stronger inhibitory activity of phillygenin than that of phillyrin. Consistent with previous mechanistic studies, these findings can be interpreted as qualitative structural hypotheses. Further validation using molecular dynamics simulations and human elastase models is necessary to confirm the stability and functional relevance of these interactions under biologically relevant conditions.

### 4.4 Mechanistic implications and biological relevance

By integrating kinetic, spectroscopic, and docking data, our findings support a model in which both phillyrin and phillygenin inhibit elastase via direct competitive occupation of the active site, accompanied by local conformational rearrangements. This dual mechanism, steric blockade combined with conformational modulation, is consistent with contemporary models of serine protease inhibition by natural phenolic compounds and provides a mechanistic bridge between enzymatic kinetics and protein structural dynamics.

### 4.5 Limitations and perspectives

Although this study provided detailed mechanistic insights into the inhibitory effects of phillyrin and phillygenin on PPE, it had several limitations. First, PPE was used as a model enzyme and further validation with human neutrophil elastase (HNE) is necessary to confirm the translational relevance of these findings. Previous studies have shown that, although PPE and HNE share high structural homology and a conserved catalytic triad, subtle differences in surface charge distribution and substrate recognition may lead to variations in inhibitor potency and binding behavior (62–64). Such enzyme-specific differences may partly explain the discrepancies in inhibitory efficacy reported across elastase studies. Second, the present study primarily focused on biochemical and biophysical characterization; aspects such as cellular uptake, metabolic stability, off-target effects, and *in vivo* efficacy or toxicity were not assessed. In contrast, some earlier investigations of plant-derived phenolic or lignan-based elastase inhibitors incorporated cellular inflammation models or animal experiments, reporting anti-inflammatory effects that extended beyond direct enzyme inhibition (24, 65, 66). The absence of biological models in the current study limits direct comparisons at the functional level, although it allows for a more rigorous dissection of the molecular mechanisms. Third, molecular docking provides a theoretical prediction of binding modes but does not capture the full dynamics of enzyme–ligand interactions. High-resolution structural studies such as X-ray crystallography and cryoelectron microscopy are required to directly verify these interactions. Notably, several previous docking-based studies relied solely on computational affinity scores without kinetic or spectroscopic corroboration (67, 68). By integrating docking with enzyme kinetics and multiple spectroscopic techniques, the present study provides a more comprehensive and experimentally supported interpretation of lignan–elastase interactions while acknowledging the inherent limitations of *in silico* modeling. Another limitation is that only two structurally related lignan compounds were investigated, which restricts the breadth of structure–activity relationships (SARs) that can be inferred. Earlier studies examining broader and chemically diverse lignan libraries have reported variable effects of glycosylation and hydroxyl substitution on elastase inhibition (16, 69, 70). Differences between these reports and the present findings may arise from variations in the lignan scaffolds, degrees of glycosylation, or experimental conditions. Here, a comparison between phillyrin and its aglycone phillygenin enables a clearer assessment of the impact of glycosylation on inhibitory potency; however, future studies incorporating a wider range of lignan subclasses are required to generalize these conclusions.

Future research should therefore aim to (i) evaluate inhibitory activity against human elastase isoforms; (ii) conduct expanded SAR analyses across structurally diverse lignans to identify key functional determinants of activity; molecular dynamics simulations are warranted in future work to validate and refine the proposed binding models; and (iii) assess anti-inflammatory efficacy, pharmacokinetics, and safety profiles in relevant cellular and animal models. Such studies would facilitate more direct comparisons with the existing literature and help reconcile the discrepancies observed among different experimental systems.

Overall, this study demonstrated that both phillyrin and phillygenin act as competitive inhibitors of porcine pancreatic elastase, with phillygenin exhibiting superior inhibitory potency. Mechanistic analyses combining enzyme kinetics, spectroscopy, and molecular docking indicated that these lignans directly engaged the catalytic pocket of elastase and induced conformational and secondary structural alterations that impaired enzymatic activity. Consistent with previous reports on phenolic elastase inhibitors, competitive inhibition and active-site targeting are common mechanistic features (71, 72). However, unlike studies suggesting the enhanced activity of glycosylated lignans, our findings indicate that the removal of the glycoside moiety enhances inhibition, likely due to reduced steric hindrance and improved accommodation within the active site. Variations in the enzyme source, assay design, and compound structural context may underlie these apparent differences.

Collectively, these results provide mechanistic insights into lignan-mediated elastase inhibition and establish a solid foundation for further exploration of its therapeutic potential in inflammation-related diseases.

## 5 Conclusion

In summary, phillyrin and phillygenin effectively inhibit elastase activity through competitive binding and modulation of enzyme conformation. Phillygenin demonstrated a markedly higher potency, highlighting the impact of structural features on inhibitory efficiency. This study not only clarifies the molecular basis of elastase inhibition by these lignans but also identifies phillygenin as a promising lead compound for the development of natural elastase inhibitors with potential applications in treating elastase-mediated and inflammatory conditions. This study provides a mechanistic framework for future SAR studies and the rational design of more potent lignan-derived elastase inhibitors.

## Data Availability

The raw data presented in the study are included in the article/supplementary material; further inquiries can be directed to the corresponding author.
